# The Novel Quantitative Technique for Assessment of Gait Symmetry Using Advanced Statistical Learning Algorithm

**DOI:** 10.1155/2015/528971

**Published:** 2015-02-02

**Authors:** Jianning Wu, Bin Wu

**Affiliations:** ^1^School of Mathematics and Computer Science, Fujian Normal University, Fuzhou 350007, China; ^2^Hospital of Fujian Normal University, Fuzhou 350007, China

## Abstract

The accurate identification of gait asymmetry is very beneficial to the assessment of at-risk gait in the clinical applications. This paper investigated the application of classification method based on statistical learning algorithm to quantify gait symmetry based on the assumption that the degree of intrinsic change in dynamical system of gait is associated with the different statistical distributions between gait variables from left-right side of lower limbs; that is, the discrimination of small difference of similarity between lower limbs is considered the reorganization of their different probability distribution. The kinetic gait data of 60 participants were recorded using a strain gauge force platform during normal walking. The classification method is designed based on advanced statistical learning algorithm such as support vector machine algorithm for binary classification and is adopted to quantitatively evaluate gait symmetry. The experiment results showed that the proposed method could capture more intrinsic dynamic information hidden in gait variables and recognize the right-left gait patterns with superior generalization performance. Moreover, our proposed techniques could identify the small significant difference between lower limbs when compared to the traditional symmetry index method for gait. The proposed algorithm would become an effective tool for early identification of the elderly gait asymmetry in the clinical diagnosis.

## 1. Introduction

The symmetry in human gait is commonly considered as one of the important features of the behavior during walking, and it is often viewed as a vital indicator of gait function of healthy individual in both clinical and research setting [1, 2]. As we have known, the gait symmetry is usually assumed as the identical function of locomotion between left and right sides of body and its change (i.e., gait asymmetry) can be found by examining the significant difference of activity between two sides such as lower limbs. The accurate evaluation of gait symmetry has an important role in the assessment of motion function [3, 4].

In nearly twenty years, although there are debates over gait symmetry based on the different definition or methods, the studies on techniques for quantitative analysis of gait symmetry have been increasing attention [3–5]. The common methods for quantifying the gait symmetry, according to the literatures reported, mainly include two classes: algebraic indices and statistical techniques [1, 3, 5]. The basic idea of these algorithms is to find the significant difference between left and right limb during walking by quantitative analysis of the gait variables [1, 3]. For example, the symmetry index (SI), the classical algebraic algorithm proposed by Robinson, is defined as

(1) $$\begin{array}{c} \text{SI}=\frac{2\left(X_{R}-X_{L}\right)}{\left(X_{R}+X_{L}\right)}\times 100\%, \end{array}$$

where *X*_*R*_ and *X*_*L*_ are the values of the measured gait parameters from the right and left limbs, respectively [3]. That is, the gait symmetry can be available when the value of SI is equal to zero by calculating the gait variables at discrete time point during gait cycle. Since the traditional SI value is obtained only by a simple average calculation, there exists the loss of the useful information associated with symmetry in gait variables. Recently, the techniques of the development of SI have been presented in publication related to gait symmetry [6]. More recently, a related study showed that the SI value is developed as the integral of the absolute value to evaluate the difference between left and right lower limb. In this study reported, the developed SI value is obtained by calculating the data set from the entire stance phase in order to provide more useful information about gait symmetry. In addition, the statistical technique has been widely applied in the gait analysis to quantify gait symmetry [4]. Most of the early studies tried to use some simple statistical techniques (such as the paired *t*-tests) to evaluate the difference between lower limbs during walking based on kinematic or kinetic gait data. These simple statistical techniques only provide limited information related to the assessment of gait symmetry [7, 8]. Recently, multivariate statistical techniques have attracted growing attention in the field of the quantization of gait symmetry in order to gather more information on insight into gait complexity [4]. Principal components analysis (PCA), as a powerful multivariate statistical technique, has been successfully applied to quantify gait symmetry in some studies. These authors have proved great potential of use of PCA for the assessment of symmetry in gait, but they also found that the selection of principal components in PCA mainly depends on the rotational criteria, in which there exists limitation of PCA in reality application such as clinical environment circumstance [9, 10]. As we have known, the complex dynamic of gait is an important characteristic of locomotion during walking, and the recorded gait data contains the “interesting” characteristic information related to the intrinsic nonlinear dynamics of human gait [1, 2, 10]. It is vital to capture those pieces of intrinsic “interesting” information from gait data for quantifying gait symmetry. However, the current technique such as the new developed SI or PCA for the quantization of symmetry in gait cannot provide the intrinsic nonlinear dynamic information which resided in gait data because they only satisfy linear algorithm [4, 10]. So, it motivates us to find the more effective method that can gain more information about insight into complex dynamic of gait for accurately quantitative assessment of gait symmetry.

It is well-known that the complex dynamic in human gait arises from the high interaction between central nervous system and various muscles [2, 3]. Gait symmetry or asymmetry can be considered the degree of “order” or “disorder” of a dynamic system. According to statistics theory, we assume that the degree of intrinsic change in dynamical system of gait is associated with the different statistical distributions; that is, it is desirable to search for the corresponding probability distribution associated with the degree of disorder in the dynamic system of gait such as gait symmetry [10]. In this study, all gait variables from the two sides of body are assumed to be independent and identically distributed. These variables satisfy the same probability distribution in the hypothesis space when the gait is symmetrical; in other words, the gait symmetry is destroyed when the entire gait variables arise from the different probability distribution. Therefore, based on the difference of probability distribution related to gait variables between lower limbs, we tried to investigate the application of the classification technique to find the small significant difference between left and right sides; that is, the advanced classification algorithm is used to recognize the difference between left and right gait, which can quantitatively assess gait symmetry.

Recently, some advanced machine learning algorithms have been more and more attractive in the gait analysis for gait classification. Practically, support vector machine (SVM), which is a novel statistics learning algorithm based on the Vapnik-Chervonenkis (VC) theory and structural risk minimization (SRM), has been widely and successfully applied in gait classification for assessment of change of gait function [11–14]. In this study, we considered that the determination of deviations from symmetry in gait is considered a binary classification task, and SVM with superior classification performance was developed to quantify gait symmetry. In order to test the effectiveness of our proposed technique, we obey the fundamental assumption that the normal or healthy gait is symmetric and pathological gait is asymmetric. Here, we acquired the kinetics gait data of lower limb of subjects from 60 subjects. In order to capture more information related to dynamic of gait from the recorded data, we defined all values of gait parameters measured during a gait cycle as a gait pattern. SVM was developed to classify the gait pattern between the right and left sides of lower limb in order to effectively determine the difference of similarity between the corresponding gait patterns. In addition, with the same gait data, we also compared our proposed technique to the traditional SI method, further suggesting that our proposed technique is more effective than traditional quantization of method for gait symmetry.

This paper was organized as follows.  Section 2 presented the procedure of kinetics gait data acquisition. In Section 3, we particularly introduced our proposed algorithm for quantifying the gait symmetry or asymmetry. In Section 4, the experimental results of this study were given. Discussions and conclusions were given in Sections 5 and 6, respectively.

## 2. Kinetics Gait Data Acquisition

In this study, the acquisition of kinetic gait data was performed with 60 healthy participants (mean age: 64.1 ± 2.35 years; height: 169 ± 5.3 cm). All participants had no known injuries or abnormalities that affect their gait. They were asked to walk at a self-determined pace on the 10 m long straight laboratory walkway where a strain gauge Bertec force platform (Bertec Corporation, Canada) was embedded in the middle. In this experiment, the foot was required to entirely step on the force platform and not to contact the edge of the platform. Prior to acquisition of data, each participant was given 20 minutes to be familiar with the data collection procedure and setting, and body weight of each participant was recorded. The sampling frequency of force platform was set to 400 Hz. For each participant, we recorded 10 successful trials from the right and left sides, respectively. In order to avoid the individual difference of participants, all recorded values of foot-ground force (GRF) were normalized by participants' bodyweight while they were further normalized with respect to the duration of the gait cycle of each limb [15–17].

## 3. SVM Classification Algorithm for Evaluating Gait Symmetry

SVM [11], a prevailing statistical learning algorithm for machine learning, is proposed by Van pick based on the Vapnik-Chervonenkis (VC) theory and structural risk minimization (SRM). Compared to traditional machine learning algorithm such as artificial neural network, SVM can yield superior generalization for new data classification because of its ability to minimize both structural and empirical risk. The basic idea of SVM for classification algorithm is to firstly map input data into a higher dimensional feature space via kernel function and then to construct an optimal separating hyperplane between the two classes in the mapped space. In this study, we assume that a gait data set *M* of points in a *n*-dimensional space belongs to two different classes +1 and −1. Here, classes +1 and −1 represent the right and left side gait pattern, respectively:

(2) $$\begin{array}{c} M=\left\{\left(x_{l},y_{l}\right),y_{l}\in \left(+1,-1\right)\right\}, \end{array}$$

where *l* ∈ {1,…, *L*}, *x*_*l*_ ∈ *R* ^*n*^.

The task of SVM is to find a function that maps the points from their data space to their label space by the following equation:

(3) f:Rn⟶+1,−1,xl⟶yl, yl∈+1,−1.

Therefore, with kernel function, the optimal separating hyper-plane can be found in the higher-dimension space, and it is expressed as [18]

(4) $$\begin{array}{c} f\left(x\right)=\mathrm{sign}\left(\underset{i\in \text{SV}}{\sum }\beta_{i}y_{i}K\left(x_{i},x\right)+b\right), \end{array}$$

where *K*(*x*_*i*_, *x*) is kernel function that satisfies Mercer's conditions; *b* is a bias estimated on the training set; *β*_*i*_ are the coefficients of the generalized optimal separating hyper-plane, and they can be obtained by solving the following quadratic programming (QP ) problem [19]:

(5) min⁡ Wβ=−βTI+12βTMβ subject  to βTy=0, βi∈0,C, Ii=1.

Since the gait data are not linearly separable in this study, in order to minimize classification error, some nonnegative slack variables (*ξ*_*i*_ ≥ 0 is a measure of misclassification errors) and a penalty function *F*_*γ*_(*ξ*) = ∑_*i*_ *ξ*_*i*_^*γ*^ *γ* ≥ 0 are employed in SVM classification algorithm. Thus, the problem of searching for the generalized optimal separating hyperplane can be considered the solution of the following optimization problem [11]:

(6) min⁡ 12w2+C∑iLξi subject  to yiwTx+b≥1−ξi,vvvvvvvvvvvvivvvvvi=1,…,L,

where *w* is the weight vector in the generalized optimal separating hyperplane. Minimizing (1/2)‖*w*‖^2^ is equal to maximizing the margin, and minimizing *C*∑_*i*_^*L*^ *ξ*_*i*_ can obtain the minimization of the classification error. *C* denotes the misclassification penalty parameter which controls the trade-off between the maximum margin and the minimum error and must be set to a given value [11].

In addition, the performance of SVM classification algorithm mainly relies on the selection of kernel functions because these functions determinate the nature of the decision surface between separated gait data [19]. In this study, the following kernels functions were used:

1. linear: (*x*_*i*_, *x*_*j*_) = *x*_*i*_ · *x*_*j*_,
2. polynomial (poly): *K*(*x*_*i*_, *x*_*j*_) = ((*x*_*i*_ · *x*_*j*_) + 1)^*d*^, where *d* is the polynomial's degree,
3. Gaussian radial basis function (RBF): *K*(*x*_*i*_, *x*_*j*_) = exp⁡(‖*x*_*i*_ − *x*_*j*_‖^2^/2*σ* ^2^), where *σ* is the width of RBF function.

As a result, we can discriminate the small difference of similarity between lower limbs by developing SVM classification model to recognize the right-left gait patterns accurately.

## 4. Experimental Results

### 4.1. The SI-Based Methods for Gait Symmetry

**Figure 1:**
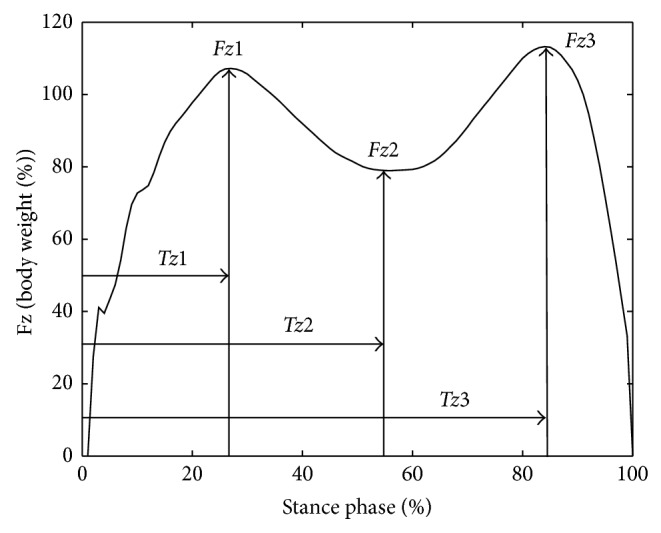
The selected six gait parameters in the vertical directional foot-ground reaction forces.

Since the variability of the forces in vertical direction is lower than that of the other direction force [18, 20], in this study, we selected the GRF in vertical direction for the quantization of gait symmetry. To more effectively evaluate our proposed technique, we firstly used the traditional SI-based method to quantify the gait symmetry of subjects. The six important gait parameters in analysis of normal gait function (three peak forces *Fz*1, *Fz*2, and *Fz*3 and their corresponding time *Tz*1, *Tz*2, and *Tz*3), as shown in Figure 1, were chosen to quantitatively assess the deviation from the gait symmetry [18, 21, 22].

At first, the variability of six gait parameters selected was estimated by using coefficient of variation (CV).

Here, for each gait parameter corresponding to right and left side of body, respectively, its value of CV can be obtained by calculating the measurement of 10 trails according to the following definition of CV:

(7) $$\begin{array}{c} \text{CV}=\frac{\text{SD}}{\text{M}}\times 100\%, \end{array}$$

where SD and M denote standard deviations and mean value, respectively. The variability of each gait parameter can be regarded as an acceptable level when CV ≤ 12.5%. In this study, the absolute symmetry index (SI) was used to evaluate the deviation from gait symmetry [13, 21–23], and it is defined as

(8) $$\begin{array}{c} \text{SI}=\left|\frac{2\left(X_{R}-X_{L}\right)}{\left(X_{R}+X_{L}\right)}\right|\times 100\%. \end{array}$$

When the SI value is less than 10%, the deviation from gait symmetry can be considered an acceptable level. In addition, we also employed a paired *t*-test technique to further test the left-right difference of each gait parameter [21, 22]. In the experiment, the significance level value (*P*) was set as 0.05; that is, when *P* ≤ 0.05, the significance of right-left difference is an acceptable level. The experimental results were presented in Table 1. From Table 1, we can see that the variability of each gait parameter selected is acceptable level due to the fact that each corresponding CV value is less than 12.5%. The symmetry in gait of subjects would seem to be an acceptable level as the obtained ASI values are less than 10%. However, there exists small difference between right and left limbs based on the obtained significance level value (*P* ≤ 0.05) from *Tz*1, *Fz*2, and *Tz*3, respectively. These results suggested that the traditional SI-based method could not discover the more intrinsic dynamic information hided in gait variables.

### 4.2. SVM for Gait Symmetry

In order to capture more information related to dynamic of gait from the recorded data, all values of gait parameters measured during a gait cycle were defined as a gait pattern; namely, each gait pattern is denoted as 101 dimensions vector according to sampling at each 1% within a normalized stance phase. These defined gait patterns were used as the input of SVM train sets to develop the SVM classifier for recognizing the right-left gait pattern.

In this study, since the number of subjects is 60, the total of gait samples including right and left side are 120. With small sample data, the cross-validation technique was used to develop and evaluate the SVM classifier [16–20]. Here, a six-fold cross-validation scheme was proposed; that is, 120 sample data including 60 right-side and 60 left-side gait sample data were divided into six segments, and each segment must consist of 10 right-side and 10 left-side gait patterns. Namely, each of the six cross-validation test segments contained 10 right-side and 10 left-side gait patterns while their respective training segment included the remaining 50 right-side and 50 left-side gait patterns. Firstly, 5 out of the 6 segments were used to train and construct the SVM decision surface while the remaining one was used in testing, and an average classification result was obtained from the testing set of the subjects' gait data. Secondly, the above procedures were repeated for 10 times. Finally, the six classification results were averaged to obtain a final performance result [12–14].

In this experiment, three common kernels (polynomial, linear, and RBF kernel) were adopted for SVM. The training SVM classifier mainly includes initialization of the training set and optimization of parameter such as regularization parameter *C*, kernel parameter *σ*, and *d* of SVM. The detailed training procedure is as follows.

**Step 1 .**

Initiate the kernel parameters such as *C*, *σ*, and *d*, and construct an initial training set.

**Step 2 .**

The optimal generalization performance of SVM was produced by adjusting the optimal parameters *C*, *σ*, and *d* based on the proposed six-cross-validation method.

In addition, accuracy, sensitivity, and specificity were used to evaluate the performance of the SVM classification, and they were defined as the following, respectively:

(9) Accuracy=TP+TNTP+FP+TN+FN×100%,Sensitivity=TPTP+FN×100%,Specificity=TNTN+FP×100%,

where TP denotes the number of true right-side gait patterns that SVM correctly identifies. FP is the number of false right-side gait patterns recognized; that is, SVM detects a right-side gait pattern as left-side. FN is the number of SVMs for the identification of a left-side gait pattern as right-side, and TN is the number of true left-side gait patterns that SVM correctly recognizes. Therefore, accuracy is defined as the ratio of the number of gait patterns accurately identified to the total of gait patterns in the tests. Sensitivity is defined as a rate of SVM for the test of ability to detect right-side gait patterns correctly, while specificity is defined as a rate of SVM for recognizing left-side gait patterns correctly.

As we have known, the superior generalization performance of SVM for classification, to the great degree, depends on the extraction or selection of the gait features as the input of the training sets. To further evaluate the classification performance of SVM, we used different methods for obtaining some good gait features from the originally defined gait pattern including 101 dimensions vector: (1) selection of the six vital gait parameters (*Fz*1, *Fz*2, *Fz*3, *Tz*1, *Tz*2, and *Tz*3) as a gait feature vector and (2) extraction of some gait feature vector from a gait pattern by PCA. Therefore, the above features vector obtained, respectively, were defined as a new gait pattern for developing SVM classifier, and the classification performance was tested by the same six-fold cross-validation design. The best results of these proposed SVM classifiers were given in Table 2. From Table 2, we could see that the classification performance of SVM reaches maximum (accuracy: 0.90, sensitivity: 0.90, and specificity: 0.88) when the some gait features obtained by PCA were used as input of SVM. Meanwhile, when the initially defined gait patterns (101 dimensions vector) and the six important gait features selected, respectively, were used as the inputs of SVM, their best result of SVM classification is almost the same (i.e., accuracy: 0.86-0.87, sensitivity: 0.85-0.86, and specificity: 0.85). These results showed that the extraction of gait feature by PCA can reduce more redundancy information which resided in gait pattern for improving the performance of SVM classification. The selection of six gait features could lose useful information about gait symmetry while the initially defined gait pattern could contain more redundancy information, which can destroy the classification performance of SVM. In this experiment, the generalization performance of SVM with nonlinear kernel function is superior to that of SVM with linear kernel function, suggesting that SVM with nonlinear kernel could obtain the more intrinsic nonlinear dynamic information from gait variables than SVM with linear. More importantly, the results of sensitivity and specificity from these proposed SVM for gait classification algorithms are slightly different, which showed that our proposed SVM algorithm could gain probability distribution of right and left side gait pattern in the mapped higher-dimension feature space. In general, these results demonstrated that, with our data, the proposed technique for gait classification could determine the small deviation between left and right lower limbs of subjects, suggesting that SVM could be used as effective tool for quantifying the gait symmetry.

## 5. Discussions

The experiment results demonstrated that, with our gait data, the SVM was able to exactly determinate the difference between probability distribution from gait pattern of right and left sides in the higher-dimension features space mapped via kernel function, and SVM for gait classification method has superior ability to quantify gait symmetry when compared to the traditional SI-based technique. Currently, the evaluation of symmetry in the able-bodied gait has received more attention in the field of biomechanics and computer science research, and it has been becoming more and more challenging endeavor [4, 6]. In this study, we chose the elderly subjects for the evaluation of gait symmetry based on the fact that gait asymmetry is produced due to the neurological and physiological changes related to the gait and aging [22–24]. To capture more useful information associated with the complex dynamic of gait, the acquisition of the kinetic gait data of subjects was performed because these acquired data satisfy theory of “kinetic chain” and avoid the limitations in the marker-based kinematics data collection such as its high cost, limited access to experiment participants, and narrowed marker positions. As showed in Table 1, the variability of all gait parameter satisfied the acceptable level because of CV ≤ 12.5%. Although the SI values from each selected gait parameter satisfied an acceptable level (i.e., <10%), there exists significant difference between some selected gait variables from left-right side such as *Tz*1, *Fz*2, and *Tz*3 according to the fact that their corresponding significance level value *P* is less than 0.05. These results demonstrated that the collected gait data contain more useful information related to the complex dynamic of gait, but the traditional SI-based technique cannot discriminate the small difference between the right-left sides of lower limb [10]. The possible reason is that the traditional SI-based methods could not capture the “interesting” information embedded in the gait data for quantifying gait symmetry.

In this study, the aim of the use of the SVM with advanced statistical learning algorithm for gait symmetry is to mine the more intrinsic nonlinear dynamic information hidden in gait data from the inter-relationship between multiple gait variables, which can accurately identify the small difference between the right-left sides of lower limbs. From Table 2, we can see that SVM classification technique can recognize the right-left gait pattern with better generalization performance. In particularly, PCA-based SVM could obtain the best generalization performance in all proposed SVM methods. Here, PCA was adopted as a preprocessing tool of gait data for reducing the redundancy information between multiple gait variables before SVM classification. Thus, we could use PCA to extract more gait features containing the more account of separation information between the separable classes [4, 13, 14]. In the experiment, we expect to obtain some good features providing additional discrimination information for improving the performance of classification because more additional extraction features do not provide all the necessary information for gait classification. The choice of the number of gait features extracted by using PCA has an important impact on the identification results. From Table 2, we can also see that when the 101 dimensions gait variables or six gait parameters selected were, respectively, used as gait feature vector (i.e., inputs of SVM), their classification performance of SVM was less than that of PCA-based SVM. The possible reason is that 101 dimensions gait vector could contain more redundant information and the selected gait features could not provide additional discrimination information between left-right lower limbs, which can deteriorate the classification performance of SVM [11, 19]. Similar dependence of classification performance on the selected features has been reported in applicability to the classification of gait patterns using various machine classifiers [12–14]

In addition, it is very important to select kernel function in the proposed SVM algorithm [19] because these kernel functions can reflect the intrinsic change in dynamical system of gait by the inter-relationship between multiple gait variables in the mapped higher-dimension feature space. In this study, considering that there may exist probability distribution of all gait variables in the higher space, we selected three kernel functions (RBF, poly, and linear kernel) for gait data analysis. From Table 2, we could find that these selected kernel functions perform well, but the generalization performance of SVM with nonlinear kernel such as RBF and Poly is superior to that of SVM with linear kernel. This is because these selected nonlinear kernels could discover “interesting” information about the interaction of all gait variables in a complicated nonlinear fashion, which can offer corresponding probability distribution associated with the degree of disorder in the dynamic system of gait. Thus, nonlinear kernel can provide more useful information related to nonlinearly separable gait patterns than linear kernel, which effectively improve the generalization performance of SVM, and determine similarities or dissimilarities between the lower limbs for quantifying gait symmetry. More important, according to the experiment results in this study, we found that our proposed statistical learning algorithm such as SVM for classification of gait pattern could accurately discriminate change of elderly gait symmetry, suggesting that the early identification of elderly gait asymmetry may be gained for the assessment of at-risk gait and prevention fall in the clinical applications [25, 26].

In this study, we also observed that the selection of the optimal parameter was vital for classification performance. The optimal parameters, such as misclassification penalty parameter *C*, and the kernel parameters (*d*, *σ*) of SVM, must be carefully selected to obtain the best classification performance. This is because the optimal value of each parameter could vary with different values of other parameters and each was chosen by using the experiment method [12, 13].

## 6. Conclusion

In this study, with our gait data, the proposed statistical learning algorithm such as SVM classification algorithm for gait symmetry can effectively capture more useful information related to complex dynamic of gait from all gait variables during gait stance and determinate the small difference of similarity between lower limbs when compared to the traditional SI-based methods. Furthermore, our proposed technique can also be applied in the other kind of gait data such as kinematics and accelerometer data to evaluate the effectiveness of gait symmetry. The proposed technique could be developed as a novel method for quantifying the gait symmetry, which would be used as the tool for the early identification of the elderly gait asymmetry in the clinic applications.

In addition, we also found that the obtained nonlinear feature information associated with gait change could contribute greatly to gaining deeper insight into the process of intrinsic nonlinear dynamics of gait. In the future work, we will focus on searching for the more robust statistical learning algorithm for gaining gait nonlinear information to evaluate the change of gait symmetry in pathologies and physiological mechanisms.

## Figures and Tables

**Table 1:** The results of statistics analysis for the selected right-left gait parameters from all subjects.

Parameters	Left-side		Right-side		SI (%)	*P* (%)
	CV (%)	Mean	CV (%)	Mean		
Fz1	8.38	113.84	8.95	114.86	2.45	3.78
Tz1	10.14	23.45	8.16	22.38	4.56	9.26
Fz2	6.42	74.39	7.38	76.56	4.35	2.34
Tz2	5.34	48.93	5.43	50.98	3.23	4.41
Fz3	9.45	110.43	7.75	112.24	2.67	6.57
Tz3	5.13	77.05	3.76	78.76	0.83	6.38

**Table 2:** Comparison of results from the different SVM classification algorithm designed.

Classifiers	Kernel	(ACC, SEN, SEP)
Extracted features-SVM	Poly	(0.90, 0.88, 0.88)
	RBF	(0.90, 0.90, 0.88)
	Linear	(0.89, 0.87, 0.87)

All variables-SVM	Poly	(0.85, 0.85, 0.83)
	RBF	(0.86, 0.85, 0.85)
	Linear	(0.83, 0.83, 0.85)

Six variables-SVM	Poly	(0.86, 0.85, 0.85)
	RBF	(0.87, 0.86, 0.85)
	Linear	(0.85, 0.85, 0.83)

Note: ACC, SEN, and SEP denote accuracy, sensitivity, and specificity, respectively.
